# Incidence and Severity of Nurse‐Sensitive Adverse Events in Older Adults After Physical Trauma: A Medical Record Review

**DOI:** 10.1111/jocn.70399

**Published:** 2026-06-16

**Authors:** Hanna Järbrink, Kristofer Bjerså, Hanna Falk Erhag, Jörgen Lundälv, My Engström

**Affiliations:** ^1^ Department of Surgery, Institute of Clinical Sciences, Sahlgrenska Academy University of Gothenburg Gothenburg Sweden; ^2^ Department of Surgery Sahlgrenska University Hospital Gothenburg Sweden; ^3^ Family Medicine, School of Public Health and Community Medicine, Institute of Medicine, Sahlgrenska Academy University of Gothenburg Gothenburg Sweden; ^4^ Primary Care, Närhälsan Majorna, Västra Götalands Regionen Gothenburg Sweden; ^5^ Institute of Health and Care Sciences, Sahlgrenska Academy University of Gothenburg Gothenburg Sweden; ^6^ Centre for a DEMentia Friendly Society With A Person‐Centred Mindset (DEMSAM) University of Gothenburg Gothenburg Sweden; ^7^ Department of Acute Medicine and Geriatrics Sahlgrenska University Hospital Gothenburg Sweden; ^8^ Department of Social Work University of Gothenburg Gothenburg Sweden

**Keywords:** aged, frailty, gerontology, healthcare‐associated infections, hospitalisation, medical record review, nursing care, nursing‐sensitive adverse events, trauma, trigger tool

## Abstract

**Aim:**

This study aimed to investigate the incidence and characteristics of nursing‐sensitive adverse events (NSAEs) in older adults (≥ 65 years) hospitalised with traumatic injuries, and to explore associations with frailty, demographic factors, injury characteristics and hospital‐related factors.

**Design:**

NSAEs were identified through a retrospective medical record review of a prospectively collected cohort.

**Methods:**

Patients ≥ 65 years admitted with physical trauma to a Swedish level I trauma centre between 2020 and 2024 were included. NSAEs were identified using a modified trigger tool chart review. Descriptive statistics were used to determine the incidence and characteristics of NSAEs in the whole cohort. Group differences, associations and predictors were examined using exact, non‐parametric, or logistic regression methods.

**Results:**

A total of 270 trauma patients ≥ 65 years were included, of whom 25.6% had experienced at least one NSAE. The overall incidence was 38.2 events per 100 admissions, with hospital‐acquired infections being the most common type of NSAE (16.7%), followed by overdistended bladder (6.3%) and pressure injuries (5.6%). Frailty and length of hospital stay were associated with an increased likelihood of NSAEs, with hospital length of stay emerging as the strongest predictor in multivariable analysis.

**Conclusion:**

NSAEs are common among older trauma patients and are associated with frailty, injury characteristics and length of stay. Improving early risk identification and ensuring timely preventive nursing care may enhance patient safety in this vulnerable population.

**Implications for the Profession and/or Patient Care:**

The findings highlight the important role of nursing in the care of older trauma patients and the need for consistent delivery of fundamental nursing care. Strengthening clinical practices that support early identification of high‐risk patients and the timely implementation of preventive interventions may improve patient safety and outcomes in this vulnerable population.

**Impact:**

This study addresses the knowledge gap regarding NSAEs in older trauma patients, a population with increased vulnerability to adverse outcomes. The findings provide insights into the occurrence and risk factors of NSAEs in this group and highlight the importance of translating risk assessment into effective clinical action. These results may inform clinical practice and support the development of strategies to improve patient safety in trauma care for older adults.

**Reporting Method:**

This study was informed by the Standard Elements in Studies of Adverse Events and Medical Error (SESAME). The completed SESAME checklist is provided in the Supporting Information S1.

**Patient or Public Contributions:**

No patient or public contributions.

## What Does This Paper Contribute to the Wider Global Clinical Community?

- Provides novel data on the incidence and characteristics of NSAEs in older trauma patients, who represent an under‐researched and vulnerable population.
- Demonstrates that one in four older trauma patients may experience at least one NSAE, most commonly healthcare‐associated infections.
- Highlights the importance of translating risk identification into timely and appropriate nursing care to improve patient safety, with relevance for trauma care systems globally.

**Abbreviations**
AE: adverse event
AUC: area under the curve
CFS: Clinical Frailty Scale
CI: confidence interval
FoC: fundamentals of care
GCS: Glasgow Coma Scale
HAI: healthcare‐associated infection
ICN: International Council of Nurses
LOS: length of stay
NISS: New Injury Severity Score
NSAE: nurse‐sensitive adverse event
OR: odds ratio
ROC: receiver operating characteristic
SD: standard deviation

## 1 Introduction

Although ‘first, do no harm’ is a fundamental principle in healthcare, patient harm remains a significant global concern. It is estimated that approximately one in 10 patients experiences harm while receiving healthcare, and more than three million deaths occur annually due to unsafe care (World Health Organization 2023). Older adults represent a particularly vulnerable patient group, and medical advances and a decline in mortality have contributed to increased life expectancy, resulting in a rapidly growing older population (Abrams et al. 2020). This demographic shift will place additional strain on already stressed healthcare systems, further underscoring the importance of preventing avoidable harm.

Compared with younger patients, older adults experience poorer outcomes across healthcare settings, especially in emergency care, where they face increased risks of hospital admission, readmission, institutionalisation and mortality (Schuster et al. 2020). This vulnerability is further amplified in surgical contexts, where frail older adults are at greater risk of adverse events than both younger and non‐frail individuals (McEvoy et al. 2023; Zhang et al. 2024). In trauma care, these risks may be exacerbated by system‐level factors, as older patients are frequently under‐triaged and less likely to receive care in specialised centres (Horst et al. 2020). Together, these factors suggest that older trauma patients constitute a particularly high‐risk population for preventable harm, including nursing‐sensitive adverse events (NSAEs), which may be mitigated through appropriate nursing care.

## 2 Background

Within the field of patient safety, *adverse events* (AEs) are often defined as: *unintended physical injuries resulting from or contributed to by medical care that require additional monitoring, treatment, or hospitalisation, or result in death* (Griffin and Resar 2009, 5). Typical examples include medication errors, surgical complications, healthcare‐associated infections, patient falls, pressure injuries and venous thromboembolism. Several of these, such as falls, pressure injuries, infections and medication errors, are considered relevant to nurses (Kawai et al. 2022), as their occurrence is closely linked to clinical nursing care (Oner et al. 2021), thus constituting NSAEs.

AEs have been shown to be common in surgical care. In a scoping review from 2018, a median of 10% (range 2.9%–21.9%) of patients were affected by an AE, with higher rates reported in surgical specialties (Schwendimann et al. 2018). In addition, a Swedish study conducted in a surgical setting reported that approximately 15% of patients experienced an AE, of which 62% were deemed preventable. The occurrence of AEs was associated with an increase in length of stay (LOS), with affected patients remaining hospitalised an average of 7.1 days longer than those without AEs. Furthermore, older adults, particularly those aged 65 years and above, demonstrated a higher risk of AEs compared to younger patients (Nilsson et al. 2016). This elevated risk may, in part, be explained by the greater physical frailty and reduced physiological resilience commonly observed in older populations, which have been associated with poorer clinical outcomes (McEvoy et al. 2023). National data from Sweden further reinforce this pattern, indicating that the majority of deaths associated with AEs occur in individuals aged 65 years and older (National Board of Health and Welfare 2024).

Previous research has demonstrated that variations in nursing care are associated with differences in patient outcomes. Suboptimal nursing care, including missed or delayed care, has been linked to increased risks of AEs such as falls, infections and medication errors, as well as poorer patient experiences and outcomes (Ball et al. 2018). However, estimating the occurrence of NSAEs remains challenging due to methodological limitations, including variations in definitions and measurement approaches, and difficulties in attributing outcomes specifically to nursing care (Kurtzman 2010). Despite these methodological challenges, available evidence indicates that NSAEs constitute a substantial proportion of patient harm in hospital settings. Estimates suggest that up to one‐third of AEs may be attributable to nursing care (D'Amour et al. 2014). In addition, a multicentre cohort study using structured detection methods reported that a substantial proportion of hospitalised patients experience NSAEs, with approximately 19% affected in orthopaedic settings (Hommel et al. 2020). Despite this, few studies have examined the contribution of nursing care to AEs, particularly in high‐risk populations such as older trauma patients. Consequently, a critical knowledge gap remains in terms of understanding the extent and nature of NSAEs in this group. Addressing this gap is essential, as reducing preventable harm may alleviate patient suffering, shorten hospital stays, reduce healthcare costs and support safer and more sustainable healthcare systems.

## 3 Aims

This study aimed to investigate the incidence and characteristics of NSAEs in older adults (≥ 65 years) hospitalised with traumatic injuries between 2020 and 2024, and to explore associations with frailty, demographic factors, injury characteristics and hospital‐related factors.

## 4 Methods

### 4.1 Design and Definitions

In this observational study, the medical records of hospitalised patients were reviewed to identify NSAEs. Based on the definition of AEs described in the background section, NSAEs were operationalised as events involving physical harm or death related to care provided or omitted, not attributable to the natural course of the patient's underlying condition, and considered preventable or potentially preventable in line with the concept of avoidable harm in the Swedish Patient Safety Act SFS 2010:659 (Swedish Parliament 2010).

Events were classified as NSAEs only if they were judged to be influenced by nursing care and potentially preventable through appropriate nursing interventions. As there is no universally accepted definition of NSAEs, this operationalisation was used to capture events where nursing care was considered to play a contributory role.

### 4.2 Setting and Participants

The study was conducted at a tertiary care university hospital and with a Level 1 trauma centre in Sweden. Patients aged ≥ 65 years who were admitted due to physical trauma between 2020 and 2024 were prospectively included. Physical trauma was defined as injury resulting from an external force (e.g., falls, traffic‐related incidents, other accidents or assaults) requiring inpatient hospital care. Patients were included at the end of the hospital stay, prior to discharge. However, inclusion could also occur after discharge via telephone contact, in which case written information and consent forms were sent to the patient's home. In total, 270 patients were included.

Eligibility further required the ability to provide informed consent. For patients with a known diagnosis of dementia, verbal assent was accepted following consultation with a close relative who could confirm the patient's presumed desire to participate. Individuals who had died before or at the time of screening were excluded, as informed consent could not be obtained.

Patients were excluded if they were assessed as having a non‐survivable injury or a prognosis indicating that they were unlikely to survive the acute phase following the trauma (*n* = 9), had sustained an irreversible spinal cord injury (*n* = 5), were acutely confused (*n* = 92), or were otherwise unable to provide informed consent. Details regarding the inclusion process, the number of excluded patients, and reasons for exclusion are shown in Figure 1.

**FIGURE 1:**
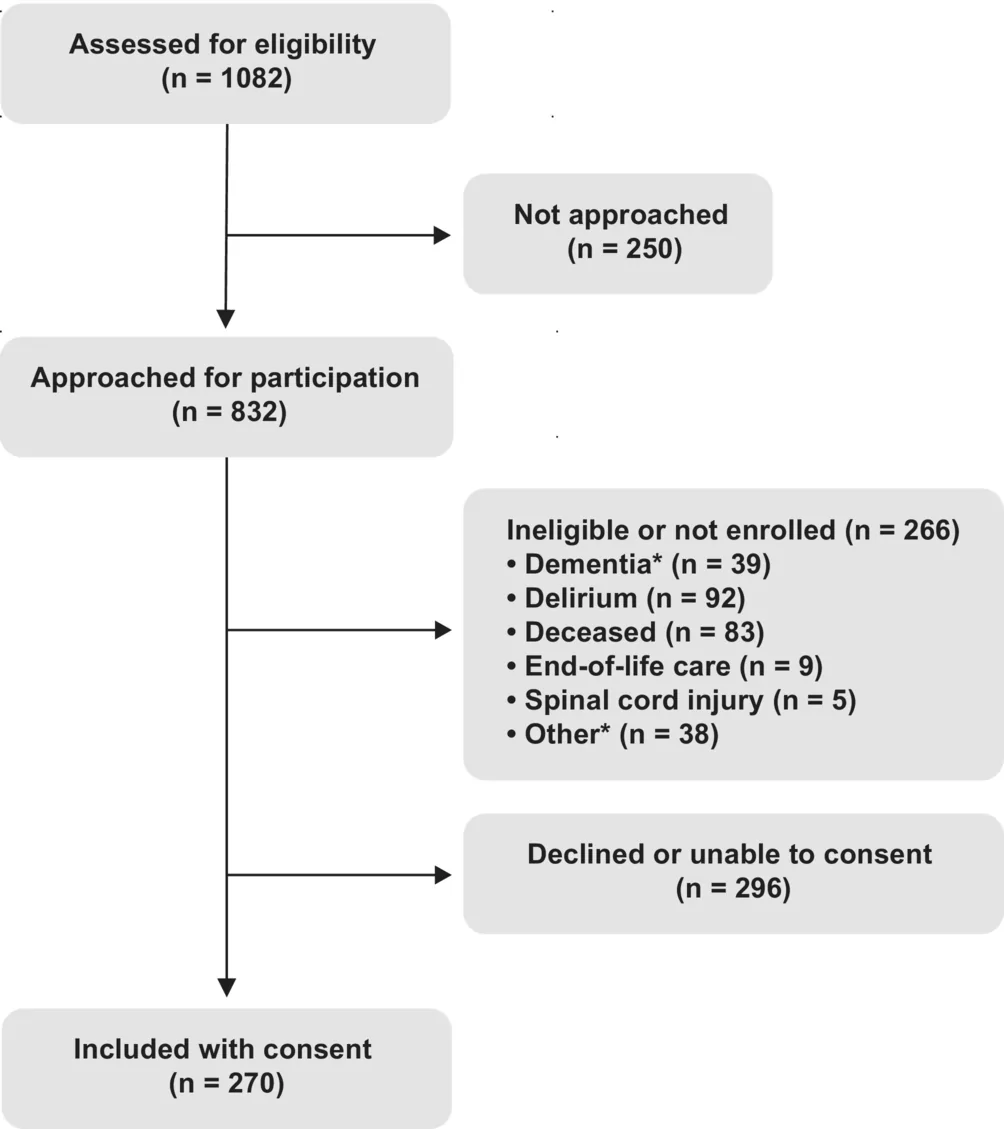
The figure illustrates the screening and recruitment process, including reasons for non‐participation and final inclusion with consent. Patients categorised as deceased had died before or at the time of screening and were therefore not approached for participation. *Dementia was an early exclusion criterion, which was changed during the study inclusion period. *Other included patients with severe mental health conditions; approaching them for study participation was deemed ethically inappropriate.

### 4.3 Data Collection

The review covered the entire hospital stay, as well as any related healthcare contacts within 30 days after discharge. To detect NSAEs, a study‐specific review protocol was developed based on the Global Trigger Tool (Griffin and Resar 2009) and the marker‐based medical record review protocol from The Swedish Association of Local Authorities and Regions (2014). The applied methodology is a modified approach that combines elements of the trigger tool and the SALAR review protocol. The protocol served as the primary guidance document during the review process, while the original Global Trigger Tool and SALAR review protocol were consulted during the protocols development and when clarification was needed.

All medical record reviews were conducted by the first author. A random sample of 27 medical records (10%) was independently reviewed by a co‐author. Interobserver agreement was 92.6% (25/27), with Cohen's kappa = 0.81. No formal training programme was undertaken. The review process was conducted without a predefined time limit, allowing sufficient time to ensure a thorough and consistent assessment of each case. To support consistency in outcome assessment, including event identification, preventability and classification, predefined criteria and structured assessment procedures were applied. To enhance validity and consistency, uncertain cases were first discussed with a senior consultant nurse, and if consensus could not be reached, a physician in the research group was consulted. All review documentation was retained for transparency.

In the first step of the review, all medical records were systematically examined to identify predefined NSAEs, as detailed in Table S1. Data extracted from the medical records also included sex, age, Clinical Frailty Scale (CFS), living situation, primary diagnosis, type of injury, New Injury Severity Score (NISS), length of stay (LOS), Glasgow Coma Scale (GCS) on admission, readmission within 30 days, level of care after discharge, type of trauma team activation, documented risk assessments (falls, pressure ulcers and urinary tract infection risk) performed within 24 h of admission, initiation of care plans for identified risk areas, occurrence and type of NSAE, day of occurrence and severity grading.

When a potential event was identified, a focused assessment was conducted to determine whether it met the criteria for an NSAE. Event identification included both acts of commission (e.g., inappropriate or harmful interventions) and acts of omission (e.g., failure to implement appropriate preventive measures). Each NSAE was recorded and analysed as a separate event. In cases of cascading events, where one event contributed to another, each event was counted individually. No formal causation scale was applied; instead, causation was assessed through clinical judgement guided by predefined criteria in the review protocol.

In this study, the NSAEs screened were classified into six predefined categories and included falls, pressure injuries (categories 2–4), overdistended bladder, medication‐related harm (i.e., medication errors or adverse drug effects), healthcare‐associated infections (HAIs) and skin or tissue injuries. Each included NSAE was recorded in terms of event definition, clinical indicators and conditions for preventability, derived from the underlying trigger tool methodologies (Table S1). Preventability was assessed through clinical judgement guided by predefined criteria in the review protocol (Table S1). Events were considered preventable if there was evidence that appropriate preventive measures had not been implemented, that identified risks were not addressed, or that care was incomplete or delayed. Preventability was assessed as part of the review process but was not analysed as a dependable outcome variable.

Severity was assessed using a modified version of the National Coordinating Council for Medication Error Reporting and Prevention (NCC MERP) Index (National Coordinating Council for Medication Error Reporting and Prevention 2001), which is also applied within the Global trigger tool methodology. Categories A–D were used as triggers to identify potential events, while severity classification was based on NCC MERP Categories E–I. Only events classified as Categories E–I, representing harm, were included in the analysis (see Table 1).

**TABLE 1:** NCC MERP categories E–I and definitions.

Category	Description
E	Temporary harm
F	Temporary harm requiring prolonged hospitalisation, outpatient care, or readmission
G	Permanent harm
H	Events requiring life‐sustaining intervention within 60 min
I	Events contributing to or resulting in death

### 4.4 Statistical Analysis

Statistical analyses were performed using SAS version 4.3. Categorical variables are presented as numbers (*n*) and percentages, and continuous variables as means with standard deviations (SDs) or medians with ranges, as appropriate. Event occurrence was reported both as the proportion of patients experiencing at least one NSAE and as the total number of events, allowing for multiple events per patient. Group comparisons between patients with and without NSAEs were conducted for demographic variables (i.e., sex and age), clinical characteristics (i.e., frailty, injury severity and primary diagnosis) and hospital‐related factors (i.e., LOS), using exact tests for categorical variables and Fisher's non‐parametric permutation test for continuous variables.

Confidence intervals (CIs) for dichotomous variables were calculated using unconditional exact limits; when exact limits were not available, asymptotic Wald confidence limits with continuity correction were used. For continuous variables, CIs for mean differences were based on Fisher's non‐parametric permutation test. Effect sizes were calculated as the absolute mean difference divided by the pooled standard deviation, reported as Cohen's d for sample sizes ≥ 50 and Hedges' g for sample sizes < 50.

Associations between predictors and NSAEs were analysed using univariable logistic regression. Odds ratios (ORs) with 95% CIs, *p*‐values, and the area under the receiver operating characteristic (ROC) curve were calculated using the original continuous values of predictors. Independent predictors were identified using stepwise multivariable logistic regression. All significance tests were two‐sided and conducted at a 5% significance level. As analyses beyond the primary descriptive aim were considered exploratory, no formal adjustment for multiple testing was applied. All analyses were conducted with the support of a professional statistical group affiliated with the hospital, ensuring methodological rigour and data confidentiality.

### 4.5 Ethical Considerations

The study was conducted in accordance with the ethical principles of the Declaration of Helsinki (World Medical Association 2024) and the guidelines of The Swedish Research Council (2024) and was approved by the Swedish Ethical Review Authority (registration numbers 766‐18, 2019‐05769 and 2020‐01194). Written informed consent was obtained from all participants prior to inclusion, and participation was voluntary with the option to withdraw at any time without explanation. Following a protocol amendment, patients with dementia were also eligible for inclusion, with proxy consent obtained from next of kin to access medical records.

## 5 Results

### 5.1 Patient Characteristics and Injury Profile

The study cohort comprised 270 patients hospitalised following a physical trauma. The mean age at admission was 78.7 years (SD 8.6; range 65–99), and 47.8% were women. Ground‐level falls were the leading cause of injury in both patients with and without NSAEs, accounting for 70.4% of all cases, with no significant difference between the groups (*p* = 0.38). Thoracic or spinal injuries were the most common primary diagnoses among patients with NSAEs (61.8%; *n* = 42). Among patients without NSAEs, thoracic/spinal injuries and intracranial injuries occurred with equal frequency (39.8%; *n* = 80); see Table 2.

**TABLE 2:** Demographic, clinical and injury‐related characteristics by occurrence of NSAE.

Variable	Total (*n* = 270)	NSAE (yes) (*n* = 69)	NSAE (no) (*n* = 201)	*p*	Mean difference (95% CI)	Effect size
Age at admission, years, mean (SD)	78.7 (8.6)	81.1 (8.5)	77.9 (8.5)	0.0073	3.25 (0.90; 5.58)	0.38
Sex, *n* (%)						
Female	112 (41.5%)	28 (40.6%)	84 (41.8%)	0.98	−1.2 (−15.6; 13.2)	0.02
Male	158 (58.5%)	41 (59.4%)	117 (58.2%)		1.2 (−13.2; 15.6)	
CFS, mean (SD)	3.70 (1.65)	4.16 (1.68)	3.54 (1.61)	0.0085	0.622 (0.174; 1.062)	0.38
Frailty status based on CFS						
Robust (CFS 1–4)	192 (71.1%)	39 (56.5%)	153 (76.1%)	0.0039	−19.6 (−33.7; −5.5)	
Frail (CFS 5–9)	78 (28.9%)	30 (43.5%)	48 (23.9%)		19.6 (5.5; 33.7)	0.42
Hospital length of stay, days mean (SD)	9.39 (9.70)	18.1 (12.2)	6.40 (6.41)	< 0.001	11.7 (9.3; 13.9)	1.42
NISS, mean (SD)	15.3 (10.9)	18.8 (12.0)	14.1 (10.2)	0.0037	4.68 (1.68; 7.56)	0.44
Grouped NISS categories						
Minor injuries ≤ 8	72 (26.7%)	13 (18.8%)	59 (29.4%)	0.0059		
Moderate injuries 9–15	76 (28.1%)	18 (26.1%)	58 (28.9%)			
Severe injuries 16–24	72 (26.7%)	16 (23.2%)	56 (27.9%)			
Critical injuries 25–74	50 (18.5%)	22 (31.9%)	28 (13.9%)			
GCS at arrival, mean (SD)*	14.7 (1.0)	14.6 (1.1)	14.7 (1.0)	0.62	−0.080 (−0.317; 0.262)	0.08
Mechanism of injury						
Ground‐level fall	190 (70.4%)	47 (68.1%)	143 (71.1%)	0.038		
Fall from height	41 (15.2%)	16 (23.2%)	25 (12.4%)			
Motor vehicle collision	16 (5.9%)	5 (7.2%)	11 (5.5%)			
Bicycle accident	13 (4.8%)	0 (0.0%)	13 (6.5%)			
Other	10 (3.7%)	1 (1.4%)	9 (4.5%)			
Grouped primary diagnosis						
Intracranial injury	95 (35.3%)	15 (22.1%)	80 (39.8%)	0.029		
Thoracic/spinal injury	122 (45.4%)	42 (61.8%)	80 (39.8%)			
Laceration	20 (7.4%)	4 (5.9%)	16 (8.0%)			
Fractures	20 (7.4%)	5 (7.4%)	15 (7.5%)			
Other	12 (4.5%)	2 (2.9%)	10 (5.0%)			
Trauma activation level						
Level 1 Highest activation	48 (18.0%)	15 (22.4%)	33 (16.5%)	0.18		
Level 2 Intermediate activation	28 (10.5%)	9 (13.4%)	19 (9.5%)			
Level 3 Lowest activation or no activation	191 (1.5%)	43 (64.2%)	148 (4.0%)			

*Notes:* Data are presented as mean (SD) for continuous variables and *n* (%) for categorical variables. For comparison between groups, Fisher’s Exact test (the lowest 1‐sided *p*‐value multiplied by 2) was used. For dichotomous variables, the Mantel‐Haenszel Chi‐square exact test was used. Ordered categorical variables: Chi‐square test for trend; non‐ordered categorical variables: Chi‐square test or Fisher’s exact test; continuos variables: Fisher’s non‐parametric permutation test. Effect size was calculatedas Cohen’s d (*n* ≥ 50) or hedges’ g (*n* < 50). Values of approximateley 0.2, 0.5 and 0.8 are considered small, medium and large effects, respectively. Abbreviations: CFS, Clinical Frailty Scale; CI, confidence interval; GCS, Glasgow Coma Scale; NISS, New Injury Severity Score; NSAE, nursing‐sensitive adverse event; SD, standard deviation.

* GCS data were avaliable ony for 250 patients.

Patients with NSAEs were significantly frailer (CFS 1–4 versus CFS 5–9, *p* 0.0039) and had a substantially longer hospital stay than the non‐NSAE group (*p* < 0.0085). Patients with NSAEs had a higher mean injury severity, as indicated by NISS, than patients without NSAEs (*p* = 0.0037). Critical injuries (NISS 25–74) were more common in the NSAE group (31.9%) than in the non‐NSAE group (13.9%) (*p* = 0.0059), Table 2.

### 5.2 Incidence of NSAE

During the study period (16/01/20 to 30/01/24), 25.6% (*n* = 69) of admissions were associated with at least one NSAE during hospitalisation. A total of 103 NSAEs were identified, corresponding to 1.49 events per affected patient (range 1–5), 40.2 events per 1000 patient days, and 38.2 events per 100 admissions. The median number of days to NSAE occurrence was 6 (range 1–67). The majority of injuries were classified as Category E (83.5%, *n* = 86). Further details are presented in Table 3. Overall, the severity of NSAEs was generally low; however, one death occurred, and in two cases life‐sustaining interventions were required.

**TABLE 3:** Distribution of NSAEs by type and severity.

NSAE type	Number of events, *n*	Median day of event (range)	Category E, *n* (%)	Category F, *n* (%)	Category G, *n* (%)	Category H, *n* (%)	Category I, *n* (%)
Total	103	6 (1–67)	86 (83.5%)	11 (10.6%)	0	5 (4.9%)	1 (1%)
Fall	4	5.5 (2–18)	3 (75%)	1 (25%)	0	0	0
Pressure ulcer	15	7 (2–40)	15 (100%)	0	0	0	0
Medication‐related harm	9	3 (1–9)	6 (66.7%)	0	0	3 (33.3%)	0
Overdistended bladder	17	1 (1–17)	15 (88.2%)	2 (11.8%)	0	0	0
Skin/tissue injury	8	8.5 (2–40)	7 (87.5%)	1 (12.5%)	0	0	0
Healthcare‐associated infection (HAI)	50	7 (1–67)	41 (82%)	6 (12%)	0	2 (4%)	1 (2%)
Pneumonia	21	5 (1–26)	17 (81%)	3 (14.3%)	0	0	1 (4.8%)
UTI	16	7 (2–16)	15 (93.8%)	1 (6.3%)	0	0	0
Wound infection	3	15 (6–16)	2 (66.7%)	1 (33.3%)	0	0	0
Candida infection	5	8 (3–21)	5 (100%)	0	0	0	0
Sepsis	5	11 (6–67)	2 (40%)	1 (20%)	0	2 (40%)	0

*Notes:* Data are presented as number (*n*) and percentage. Severity classified according to NCC MERP Categories (E–I). A single patient may have experienced more than one NSAE; therefore, the total number of events exceeds the number of affected patients. Percentages are calculated within each NSAE category.

Abbreviations: HAI, healthcare‐associated infection; NSAE, nursing‐sensitive adverse event; UTI, urinary tract infection.

HAIs were most frequent, affecting 16.7% (*n* = 45) of all the patients in the cohort and accounting for approximately 49% of all NSAE events. Among these, pneumonia was the predominant type, followed by urinary tract infection, candida infection, sepsis and wound infection (Table 3). Other commonly occurring NSAEs in the total cohort included overdistended bladder (6.3% *n* = 17), pressure ulcer (5.6% *n* = 15), medication‐related injury (3.3% *n* = 9) and skin/vessel event (2.6% *n* = 7). Fall events were least common, occurring in 1.5% of patients (*n* = 4). NSAEs occurred in 29.8% of patients who underwent risk screening, compared with 20.9% of those not screened; however, this difference was not statistically significant (*p* = 0.13). Among the 141 patients who underwent risk assessment, 91 (64.5%) had an identified risk and a corresponding care plan initiated, while 19 (13.5%) had an identified risk but no care plan was initiated. The remaining 31 patients (22.0%) were assessed as not being at risk and therefore did not receive a care plan. NSAEs occurred in 34 patients (37.4%) with an initiated care plan, compared with 4 (21.1%) among those identified being at risk without a care plan, and 4 (12.9%) among those assessed as not being at risk.

To further explore group differences, the occurrence of specific NSAEs was compared between frail (i.e., CFS ≥ 5) and non‐frail patients. Overall, a significantly higher proportion of frail patients experienced NSAEs compared with non‐frail patients. As shown in Table 4, frail patients had a significantly higher proportion of HAIs compared to non‐frail patients (26.9% vs. 12.5%, *p* = 0.0086). No statistically significant differences were observed for other NSAE types.

**TABLE 4:** Occurrence of NSAE type by frailty status.

Variable	Total (*n* = 270)	Robust (CFS 1–4) (*n* = 192)	Frail (CFS 5–9) (*n* = 78)	*p*	Difference between groups mean (95% CI)	Effect size
Healthcare‐associated infection event						
Yes	45 (16.7%)	24 (12.5%)	21 (26.9%)	0.0086	−14.4 (−26.2; −2.6)	0.37
No	225 (83.3%)	168 (87.5%)	57 (73.1%)		14.4 (2.6; 26.2)	
Overdistended bladder event						
Yes	17 (6.3%)	10 (5.2%)	7 (9.0%)	0.38	−3.8 (−11.7; 4.2)	0.15
No	253 (93.7%)	182 (94.8%)	71 (91.0%)		3.8 (−4.2; 11.7)	
Pressure ulcer event						
Yes	15 (5.6%)	7 (3.6%)	8 (10.3%)	0.072	−6.6 (−14.7; 1.5)	0.26
No	255 (94.4%)	185 (96.4%)	70 (89.7%)		6.6 (−1.5; 14.7)	
Medication‐related harm						
Yes	9 (3.3%)	8 (4.2%)	1 (1.3%)	0.42	2.9 (−1.8; 7.6)	0.18
No	261 (96.7%)	184 (95.8%)	77 (98.7%)		−2.9 (−7.6; 1.8)	
Skin/tissue event						
Yes	7 (2.6%)	6 (3.1%)	1 (1.3%)	0.70	1.8 (−2.6; 6.3)	0.13
No	263 (97.4%)	186 (96.9%)	77 (98.7%)		−1.8 (−6.3; 2.6)	
Patient fall event						
Yes	4 (1.5%)	1 (0.5%)	3 (3.8%)	0.15	−3.3 (−8.6; 2.0)	0.23
No	266 (98.5%)	191 (99.5%)	75 (96.2%)		3.3 (−2.0; 8.6)	

*Notes:* Medication‐related harm includes adverse drug reactions or medication errors resulting in patient harm, such as over‐sedation, confusion, or organ‐related effects. Data are presented as *n* (%) for categorical variables. Group comparisons were performed using Fisher's exact test. Effect size is reported as Cohen's d (*n* ≥ 50) or Hedges' g (*n* < 50).

Abbreviations: CFS, Clinical Frailty Scale; NSAE, nursing‐sensitive adverse event.

### 5.3 Factors Associated With NSAE

Univariable logistic regression analyses identified LOS, higher CFS scores, greater age, greater injury severity and certain primary diagnoses as factors associated with NSAEs (Table 5). When analysed as a continuous variable, higher CFS scores were associated with an increased likelihood of experiencing an NSAE, indicating a graded increase in risk. In addition, patients classified as frail (CFS 5–9) had a significantly higher risk of NSAE compared with robust patients (CFS 1–4). LOS demonstrated good discriminative ability (AUC = 0.85), while frailty and age showed more modest but clinically meaningful discrimination (Table 5).

**TABLE 5:** Univariable logistic regression analysis of factors associated with NSAEs.

Variable	Value	*n*/*N* (%) of event	OR (95% CI) nurse‐sensitive adverse event (NSAE)	*p*	AUC (95% CI)
LOS OR per day	1 to < 4	4/98 (4.1%)	1.17 (1.12–1.22)	< 0.001	0.85 (0.81–0.90)
	4 to < 11	13/86 (15.1%)			
	11 to 79	52/86 (60.5%)			
CFS OR per CFS point	very fit ‐ <managing well (1–2)	15/74 (20.3%)	1.25 (1.06–1.47)	0.0074	0.61 (0.53–0.69)
	managing well ‐ <mild frailty (3–4)	24/118 (20.3%)			
	mild frailty ‐severe frailty (5–9)	30/78 (38.5%)			
Frailty status based on CFS	Robust (CFS 1–4)	39/192 (20.3%)	Reference	0.0023	0.60 (0.53–0.66)
	Frail (CFS 5–9)	30/78 (38.5%)	2.45 (1.38–4.36)		
Age at admission (years) (OR per 5 units)	65 – < 74	14/87 (16.1%)	1.25 (1.06–1.46)	0.0076	0.61 (0.54–0.69)
	74 to < 84	22/98 (22.4%)			
	84 to 99	33/85 (38.8%)			
Gender	Female	28/112 (25.0%)	Reference	0.86	0.51 (0.44–0.57)
	Male	41/158 (25.9%)	1.05 (0.60–1.83)		
New Injury Severity Score (NISS) OR per NISS unit	< 9	13/72 (18.1%)	1.04 (1.01–1.06)	0.0028	0.62 (0.54–0.70)
	9 to < 16	18/76 (23.7%)			
	16 to < 25	16/72 (22.2%)			
	≥ 25	22/50 (44.0%)			
GCS at arrival	3 to 14	11/45 (24.4%)	Reference	0.99	0.50 (0.44–0.56)
	15	50/205 (24.4%)	1.00 (0.47–2.11)		
Grouped Primary Diagnosis	Intracranial injury	15/95 (15.8%)	Reference	0.035***	0.62 (0.55–0.69)
	Thoracic/spinal injury	42/122 (34.4%)	2.80 (1.44–5.45)	0.0024	
	Laceration	4/20 (20.0%)	1.33 (0.39–4.55)	0.65	
	Fractures	5/20 (25.0%)	1.78 (0.56–5.63)	0.33	
	Other	2/12 (16.7%)	1.07 (0.21–5.36)	0.94	
Trauma activation level	Level 1 Highest activation	15/48 (31.3%)	Reference	0.31***	0.55 (0.48–0.62)
	Level 2 Intermediate activation	9/28 (32.1%)	1.04 (0.38–2.83)	0.94	
	Level 3 Lowest activation or no activation	43/191 (22.5%)	0.64 (0.32–1.29)	0.21	

*Notes:* All analyses were performed using univariable logistic regression. Data are presented as *n*/*N* (%) where applicable. OR represents the change in odds per unit increase in the predictor variable. A higher AUC indicates better model discrimination, where 1.0 represents perfect discrimination, and 0.5 represents no better than chance.

Abbreviations: AUC, area under the receiver operating characteristic curve; CFS, Clinical Frailty Scale; CI, confidence interval; GCS, Glasgow Coma Scale; LOS, length of stay; NISS, New Injury Severity Score; NSAE, nursing‐sensitive adverse event; OR, odds ratio.

*** Indicates the *p*‐value for the overall effect of the variable.

Patients classified as frail (CFS ≥ 5) had more than twice the odds of experiencing an NSAE than robust patients. Thoracic or spinal injuries were associated with a higher occurrence of NSAEs than intracranial injuries. Injury severity, measured by NISS, was significantly associated with NSAEs, although with limited discriminative performance.

In the multivariable logistic regression analysis, controlling for age, sex, NISS, primary diagnosis and trauma activation level, both LOS (AUC = 0.85, *p* < 0.001) and frailty (AUC = 0.60, *p* = 0.0023) remained independent predictors of NSAEs. The final model demonstrated excellent overall discrimination (AUC = 0.87), indicating a strong ability to distinguish between patients who experienced and did not experience NSAEs.

## 6 Discussion

This study demonstrates that NSAEs are common among older adults in the trauma population. Estimates from a systematic review and meta‐analysis using trigger tool methodology indicate an incidence of approximately 30 AEs per 100 admissions in acute care settings (Eggenschwiler et al. 2022). In comparison, 38.2 events per 100 admissions were observed in the present study. However, this comparison should be interpreted with caution, as the meta‐analysis demonstrated substantial heterogeneity (*I* ^2^ = 99.7%) and a wide prediction interval (5.4–164.7 events per 100 admissions), reflecting considerable variation across study populations and methodologies. Within this context, the rate observed in the present study falls within the expected range and may reflect differences in case mix, particularly the inclusion of an older and more vulnerable trauma population.

Although studies explicitly focusing on NSAEs remain limited, existing evidence suggests that a substantial proportion of AEs may be attributable to nursing care, with estimates of around one‐third reported in hospital populations (D'Amour et al. 2014). In addition, a study using structured adverse event detection methods within an orthopaedic care context reported that approximately 20% of patients undergoing elective or acute hip arthroplasty experienced at least one nursing‐sensitive adverse event during hospitalisation (Hommel et al. 2020). While direct comparisons are constrained by differences in definitions, measurement approaches and patient populations, these findings are broadly consistent with both the high occurrence and the distribution of NSAEs observed in the present study. In line with previous research, a substantial proportion of events relate to fundamental care processes such as infection prevention, mobilisation and elimination (D'Amour et al. 2014; Hommel et al. 2020). Moreover, the observed associations with older age, frailty and severity of illness are consistent with established predictors of AEs (Eggenschwiler et al. 2022; McEvoy et al. 2023). Taken together, these findings suggest that NSAEs constitute a substantial and potentially under‐recognised component of patient harm, particularly among vulnerable populations.

In our cohort, HAIs were the most frequent NSAEs, affecting 16.7% of patients, followed by overdistended bladder and pressure injuries. This pattern is consistent with previous research identifying infections and pressure injuries as among the most common NSAEs in hospitalised patients, particularly in older and functionally impaired populations (D'Amour et al. 2014; Hommel et al. 2020; McEvoy et al. 2023). We also observed that NSAEs occurred more frequently among patients with thoracic or spinal injuries than among those with intracranial injuries, although this association did not remain statistically significant after adjustments. This pattern may nonetheless reflect clinically plausible mechanisms, as thoracic and spinal trauma often involve prolonged immobilisation, respiratory compromise and interventions such as chest drainage, all of which impair respiratory mechanics and increase the risk of hospital‐acquired pneumonia and other nurse‐sensitive complications (Alotaibi et al. 2025; Kawai et al. 2022; MacCallum et al. 2020). Reduced mobility and shallow breathing not only elevate pulmonary risks but also predispose patients to pressure injuries, urinary tract infections and thromboembolic events (Wu et al. 2018).

Early identification and continuous nursing surveillance of patients at high risk of AEs are essential for targeted prevention (Han et al. 2021). However, in the study at hand, no significant difference in NSAE occurrence was observed between patients who underwent a standardised risk assessment within 24 h of admission and those who did not, suggesting that risk identification alone may be insufficient to prevent AEs. This is consistent with previous research indicating that risk assessment does not reduce AEs unless followed by appropriate and timely preventive interventions (Hillier et al. 2025; Moore and Patton 2019; National Institute for Health and Care Excellence 2025). In the present study, a care plan was initiated for approximately two‐thirds of patients following risk assessment, while some patients did not receive a care plan despite identified risks. A higher proportion of patients with a care plan experienced an NSAE, probably reflecting confounding by indication, whereby patients at higher risk are both more likely to receive a care plan and more likely to experience AEs. Together, these findings suggest that patient safety depends not only on risk identification and planning, but on how risk information is translated into timely and appropriate clinical action. Factors such as clinical judgement, frailty, workload and the capacity to deliver fundamental nursing care are likely to influence this process. However, nurses' ability to provide such care may be constrained by administrative demands and tasks unrelated to direct patient care, often referred to as illegitimate tasks (Recio‐Saucedo et al. 2018).

The association between longer hospital stays and NSAEs probably reflects a bidirectional relationship, whereby complications both contribute to prolonged hospitalisation while extended exposure to inpatient care increases the risk of AEs. Frailty, although demonstrating limited discriminative ability on its own, remained an independent predictor of NSAEs when considered alongside other factors, supporting its clinical relevance as a marker of vulnerability (McEvoy et al. 2023). These findings indicate that risk is dynamic and requires ongoing assessment and adaptation of care, rather than reliance on single‐point screening.

To improve patient safety for older trauma patients, healthcare organisations may need to move beyond risk identification alone and ensure conditions exist that support the consistent delivery of fundamental nursing care. This includes enabling sufficient time for direct patient care and supporting clinical judgement in the management of complex and vulnerable patients.

### 6.1 Strengths and Limitations

The main limitation of this study is the incomplete inclusion of eligible patients, which may have introduced selection bias. Not all eligible patients were approached or included, and the exclusion of individuals unable to provide informed consent may have led to underrepresentation of more vulnerable patients. Recruitment was further affected by organisational constraints during the COVID‐19 pandemic, potentially limiting generalisability. However, the use of proxy consent enabled the inclusion of patients with dementia, thereby ensuring representation of a frail and under‐researched population.

In addition, the use of a predefined and modified set of NSAEs may have led to underestimation of the overall burden of events, as not all relevant AEs were captured.

As with all record review studies, findings depend on the quality and completeness of clinical documentation, which can vary in acute care settings and may contribute to the under‐detection of events. Furthermore, the use of standardised assessment tools and the heterogeneity of the study population may have introduced measurement variability and limited the ability to detect smaller associations.

A strength of the study is the use of a structured and systematic record review methodology based on the Global Trigger Tool, which enables more sensitive detection of AEs than traditional reporting systems.

### 6.2 Recommendations for Further Research

Future research should build on the findings of the present study, given the substantial burden of NSAEs observed in this vulnerable trauma population. Large multicentre studies of older trauma patients across diverse healthcare systems and national contexts, including Scandinavian countries, are needed to strengthen the evidence base and improve generalisability. In addition, intervention studies should evaluate whether systematic identification of high‐risk patients, including frailty assessment, combined with timely, tailored preventive strategies, can reduce AEs in older trauma populations. Finally, research should examine how organisational factors, such as staffing levels, workload and care delivery processes, influence the implementation of preventive measures and the occurrence of AEs in clinical practice.

### 6.3 Implications for Policy and Practice

These findings highlight the importance of nursing surveillance as a key component of patient safety in the care of older trauma patients. The identification of frailty and injury severity as factors associated with NSAEs supports the use of structured risk stratification to guide preventive nursing interventions, including infection prevention, mobilisation and ongoing clinical monitoring.

Although organisational factors were not directly assessed in this study, effective implementation of such interventions probably depends on conditions that support nursing practice, including adequate staffing, continuity of care and the ability to prioritise direct patient care. Strengthening these conditions may facilitate timely preventive actions and contribute to reducing the occurrence of NSAEs in this vulnerable population.

## 7 Conclusion

This study demonstrates that NSAEs are frequent among older trauma patients and are strongly associated with factors such as frailty, injury characteristics and LOS. The findings highlight the importance of early identification and continuous monitoring of patients at increased risk, as well as the need to ensure that risk assessment is effectively translated into timely and appropriate preventive care. Strengthening the conditions that support consistent delivery of fundamental nursing care may contribute to improved patient safety in this vulnerable population.

## Funding

This work was supported by the Region Västra Götaland, grants from the Swedish state under the agreement between the Swedish Government and the county councils, the ALF agreement, Grant No. ALFGBG‐1005718 and the Agneta Prytz‐Folke and Gösta Folke Foundation, which aims to support research on diseases associated with ageing. Open access to this study is provided by the University of Gothenburg.

## Conflicts of Interest

The authors declare no conflicts of interest.

## Supporting information


**Data S1:** jocn70399‐sup‐0001‐Supinfo.docx.

**Table S1:** Definition and assessment criteria for included NSAEs.

## Data Availability

The data that support the findings of this study are available on request from the corresponding author. The data are not publicly available due to privacy or ethical restrictions.
