# Supplementary material for: Incidence and Severity of Nurse‐Sensitive Adverse Events in Older Adults After Physical Trauma: A Medical Record Review
Source: J Clin Nurs. 2026 Jun 16;35(11):4839–49. doi: 10.1111/jocn.70399 (PMC13629271; doi:10.1111/jocn.70399)
Supplement: Supplementary file 1 [file JOCN-35-4839-s001.docx]

SESAME — Standard Elements in Studies of Adverse events and Medical Error

| *Elements* | Item # | Guidance for reporting | Reported on page # |
| --- | --- | --- | --- |
| *TITLE AND ABSTRACT* | | |  |
| *Title* | 1 | Include a commonly used term (e.g., adverse event, near-miss) that conveys the study topic in the title | 1 |
| *Abstract* | 2 | Provide an informative and balanced summary of background, objectives, methods, results and conclusion | 1-2 |
|  | 3 | Use informative wording, including keywords, to facilitate correct indexing and retrievability in bibliographic databases | 1-2 |
| *INTRODUCTION* | | |  |
| *Background/ rationale* | 4 | Describe the scientific and clinical background and rationale for undertaking the study (e.g., to characterize harm, measure the adverse event rate, test or compare a surveillance tool) | 4-6 |
| *Objectives* | 5 | State specific objective(s) | 6 |
| *METHODS* | | |  |
| *Study design* | 6a | Describe the study design (e.g., retrospective or prospective; observational or interventional) | 6 |
|  | 6b | Provide details of any patient or public involvement during any phase of the study (from design to dissemination) or state no involvement | 2 |
| *Setting details* | 7a | Describe the country/countries in which the study takes place or that is the source for data used | 7 |
|  | 7b | Describe the setting (e.g., academic, community, both, urban, rural) | 7 |
|  | 7c | Describe the facility or unit type (e.g., hospital, ambulatory, intensive care unit, nursing home) and volume (e.g., single or multi-center, number of inpatient beds, annual visit volume, catchment area) | 7 |
| *Population details* | 8a | Describe any eligibility criteria related to a target medical condition or specialty (e.g., oncology, orthopedic, acute/elective care) | 7 |
|  | 8b | Describe any eligibility criteria related to participant demographics (e.g., adult, age, race, ethnicity, gender) | 7 |
| *Exclusion criteria* | 9 | Describe any exclusion criteria for participants or events | 8 |
| *Case finding/ sampling strategy* | 10a | Provide details for the study period (e.g., dates, recruitment period, duration) | 7 |
|  | 10b | Describe the method of case finding used (e.g., all records meeting screening criteria; triggered records, computerized surveillance) | 7 |
| *Reviewers and training* | 11a | Describe reviewer recruitment, qualifications, and experience in event adjudication | 11 |
|  | 11b | Describe reviewer training and what performance standards for reviewers (if any) were required prior to independent review | 11 |
| *Review process* | 12a | Describe how reviews were conducted (e.g., by an individual, multiple reviewers working independently or in phases, or together as a group) and any consensus technique used | NA |
|  | 12b | Describe any time limit on the duration of reviews, as this may impact outcome estimates (e.g., a maximum of 20 minutes per review) | 11 |
|  | 12c | Describe how individual events were determined (e.g., implicit or explicit review, use of an algorithm, use of automated/machine learning approaches) | 9 |
| *Event scope and definition* | 13a | Indicate the outcome(s) of interest (e.g., all-cause harm, preventable or ameliorable harm, errors, near misses, non-harm events) and Include explicit definitions for the outcome(s) of interest. If harm is the outcome, indicate whether the definition of harm requires that an intervention take place. | 9 |
|  | 13b | Describe the causation standard used when determining events (e.g., "caused by" vs. "resulting from or contributed to by" health care) and specify whether a scale or rating system was used to characterize the confidence of causation and if so, describe this and its origin. | 9 |
|  | 13c | Provide the time frame(s) for event inclusion (e.g., present on arrival, during the index visit, some period of time after an intervention) | 7 |
|  | 13d | Indicate whether event capture included acts of omission, acts of commission or both | 10 |
|  | 13e | Indicate whether “cascading” events, where one event caused the next, were counted as a single event or each event was counted separately | 10 |
| *Characterization of events* | 14a | Describe the taxonomy or classification system used to describe the event and/or contributing factors (e.g., surgical/procedural events, health care-acquired infections, adverse drug events) | 10 |
|  | 14b | Describe whether a severity assessment was made and if so, what scale was used and its origin | 11 |
| *Preventability* | 15a | Indicate whether preventability was assessed and if so, provide the explicit definition used | 10-11 |
|  | 15b | Describe any preventability scale or rating system used, its origin and the language used for various ratings (e.g., definitely not preventable, possibly- vs. potentially preventable) | NA |
|  | 15c | Describe the reviewer process used in determining preventability (e.g., single or tiered review, single or dual reviewers per tier, group review) | 9-10 |
| *Quality assurance* | 16a | Indicate any deviations from the intended study plan | NA |
|  | 16b | Describe any quality assurance mechanisms to improve validity and reliability (e.g., monitoring/auditing, feedback provided to reviewers) | 9 |
|  | 16c | Include assessment(s) of interrater reliability for outcomes in the study (e.g., whether an event represented harm, its preventability and categorizations) and describe how assessments were conducted and calculated | 9 |
|  | 16d | For any automated electronic data capture describe whether there was any process to validate data against manual review | NA |
| *Data analysis* | 17a | Describe all statistical methods used, including whether this study includes a post-hoc analysis and how missing data were handled | 12-13 |
|  | 17b | Explain how the sample size was determined if causal inferences are implied | 7 |
|  | 17c | Specify the unit(s) used for any presentation of event rates (e.g., number of events, number of events per patient, proportion of visits with one or more events, number of events per visit/ hospitalization, events per 100 visits/ hospitalizations or per 1000 patient days) | 12 |
|  | 17d | Indicate whether any scales that were used to assess causation or preventability were collapsed for analysis | NA |
|  | 17e | Describe procedures used to assess and control for bias or confounding | 13 |
| *RESULTS* | | |  |
| *Participants* | 18 | Report the numbers of participants at each stage of the study - those eligible, excluded (with reasons for exclusion), included and analyzed, included but not analyzed. Consider including a flow diagram to depict the design and patient flow. | 8 |
| *Descriptive data* | 19 | Report data on characteristics of study participants (e.g., demographic, clinical, social determinants of health) | 14-15 |
| *Study findings* | 20 | Report the findings of study including all outcomes described in the Methods section | 14-19 |
| *DISCUSSION* | | |  |
| *Key results* | 21 | Summarize key results with reference to study objectives and discuss the findings in relation to existing literature | 19-22 |
| *Strengths and limitations* | 22 | Discuss strengths and limitations of the study including sources of potential bias, and internal and external validity | 23 |
