# Supplementary material for: Incidence and Severity of Nurse‐Sensitive Adverse Events in Older Adults After Physical Trauma: A Medical Record Review
Source: J Clin Nurs. 2026 Jun 16;35(11):4839–49. doi: 10.1111/jocn.70399 (PMC13629271; doi:10.1111/jocn.70399)
Supplement: Supplementary file 2 [file JOCN-35-4839-s002.docx]

| **NSAE** | **Definition (summary)** | **Based on methodology** | **Key clinical indicators** | **Preventability criteria (summary)** |
| --- | --- | --- | --- | --- |
| **Fall with injury** | Physical injury resulting from a fall during hospitalisation | Swedish GTT | Fall event + documented injury (e.g. fracture, hematoma, wound) | No risk assessment, lack of preventive measures, inadequate monitoring, or unaddressed medication/nutrition/environmental risks |
| **Pressure ulcer** | Category 2–4 pressure ulcers acquired during hospital stay | Swedish GTT | Documented skin breakdown ≥ category 2 | No risk assessment, no preventive interventions (e.g. repositioning, pressure relief), or inadequate follow-up |
| **Healthcare-associated infection (HAI)** | Infection occurring ≥48h after admission or shortly after discharge | GTT + Swedish GTT | Signs of infection (e.g. CRP, diagnosis, antibiotics) | Delayed recognition, lack of preventive care, prolonged catheter use, inadequate hygiene or mobilisation |
| **Medication-related event** | Harm caused by medication error or adverse drug event | GTT | Incorrect drug/dose, adverse reaction, clinical deterioration | Contraindications ignored, incorrect dosing, lack of monitoring, or inappropriate drug combinations |
| **Overdistended bladder** | Urinary retention with bladder volume ≥500 ml | Swedish GTT | Bladder scan or documented retention | Lack of monitoring, delayed catheterisation, or failure to act on urinary retention |
| **Skin injury (other)** | Care-related skin or tissue injury (e.g. vascular or device-related) | Swedish GTT | Documented skin/tissue damage | Inadequate preventive care, improper handling, or lack of monitoring |

Supplementary table 1. Definition and assessment criteria for included NSAEs.
